# The Impact of Study Mode (Online vs. Hybrid) on Early Symptoms of Depression and Coping Strategies among University Students in Poland in Time of COVID-19 Pandemic—Preliminary Study

**DOI:** 10.3390/brainsci11121578

**Published:** 2021-11-29

**Authors:** Anna Drelich-Zbroja, Anna Jamroz-Wiśniewska, Maryla Kuczyńska, Monika Zbroja, Weronika Cyranka, Katarzyna Drelich, Olga Pustelniak, Izabela Dąbrowska, Katarzyna Markiewicz

**Affiliations:** 1 Department of Interventional Radiology and Neuroradiology, Medical University of Lublin, 20-090 Lublin, Poland; maryla.kuczynska@gmail.com (M.K.); izis608@gmail.com (I.D.); 2 Department of Neurology, Medical University of Lublin, 20-090 Lublin, Poland; annajamrozwisniewska@umlub.pl; 3 Students’ Scientific Society at the Department of Pediatric Radiology, Medical University of Lublin, 20-093 Lublin, Poland; m.zbroja8888@gmail.com (M.Z.); k.drelich1111@gmail.com (K.D.); 4 Students’ Scientific Society at the Department of Interventional Radiology and Neuroradiology Medical, University of Lublin, 20-090 Lublin, Poland; weronika.cyranka@gmail.com (W.C.); olgamariapustelniak@gmail.com (O.P.); 5 Institute of Psychology and Human Science, University of Economics and Innovation in Lublin, 20-209 Lublin, Poland; katarzyna.markiewicz@wsei.lublin.pl

**Keywords:** COVID-19, depression, hybrid learning, Mini-COPE inventory, online learning

## Abstract

Introduction: mental health has been one of the most important issues surrounding the COVID-19 pandemic; mental disorders can be exacerbated by isolation during lockdowns or online learning. The aim of this study was to analyze the relationship between non-clinical (early) symptoms of depressed moods, personality traits, and coping strategies, as well as whether the learning mode (online versus hybrid) differentiates the experiences of these early symptoms and coping strategies. Methods: 114 university students aged 19 to 34, whose education model was changed from stationary to hybrid or online due to COVID-19 restrictions, participated in the study. The participants completed the online questionnaire, which consisted of two sections: (1) demographic questions to characterize the subjects and 44 questions based on the literature review. (2) Mini-COPE Inventory. Results: the study showed that the fully online study mode has a negative impact on the mental health of students; hybrid students are more likely to use active and positive coping strategies, which effectively help to control negative thoughts and/or reduce negative mental states. Conclusions: the COVID-19 pandemic has had significant psychological effects that will extend to coming years; therefore, implementing systemic psychological care is of utmost importance.

## 1. Introduction

In December 2019, when the media reported on a series of respiratory infections emerging from Wuhan, China, no one expected it would lead to a global pandemic [1]. A “pandemic” itself is nothing new. For example, humanity experienced the Spanish flu pandemic of 1918–1919, cholera in the 19th century, the plague in the 14th century, and the Antonine Plague around 500 BC [2]. These diseases decimated populations throughout the world and forced millions of people to modify the way they lives. Each pandemic had an enormous impact on the human psyche.

COVID-19, an acute respiratory infectious disease caused by the SARS-CoV-2 virus, has affected more than 243 million people, leading to almost 5 million worldwide deaths, according to the World Health Organization (WHO). In Poland, 2,982,143 people have been infected. Unfortunately, the COVID-19 disease has also led to the deaths of 76,540 Poles (gov.pl at 27 October 2021).

Due to the lack of knowledge about courses, treatments, and possible effects of COVID-19, it was necessary to implement emergency measures to prevent virus transmission. The most significant measures were “lockdowns” implemented in 82 countries (unicef.org at 27 October 2021). National borders, shopping malls, gyms, swimming pools, etc., were closed. Millions of people were required to modify their lives, for example, by engaging in remote work and online learning. Encounters with relatives, other than those of the same household, were also restricted.

This had significant socioeconomic consequences. As social interactions are the “default” modes of human communication, the pandemic will have long-term consequences, in regard to mental health and emotional well-being [3]. The anxiety of infecting oneself and family members, limited access to research and medical care, overloading of media with messages related to the pandemic, changes in daily routine, inability to develop passions, and restraints in playing sports, but most of all the isolation and limited contact with family and friends, are primary factors contributing to mental disorders. Since the pandemic, many people have experienced problems surrounding sleeping, appetite, nervousness, and attention deficit disorders.

However, in everyday life, the infection itself is the most important issue in patients and medical professionals who have direct contact with patients (doctors, nurses) or their relatives. The most important aspect is proper diagnosis and treatment of immediate respiratory symptoms of the cardiovascular, gastrointestinal, and nervous systems. SARS-CoV-2 infection can lead to long-term mental and cognitive changes, including so-called “brain fog” due to virus-associated hypoxia in some areas of the brain, which causes worsening of neuronal metabolism and mitochondrial dysfunction [4].

Relatively few research studies have described the impacts of COVID-19 on mental health. Yang Li et al. conducted a meta-analysis that included 27 studies on a population of >700,000 students, comparing the prevalence of depression and anxiety before and during the pandemic. Results indicated that it was 21% and 19% before 1 March 2020, and 54% and 37% after 1 March 2020 [5]. Furthermore, two other meta-analyses estimated a 35% prevalence of anxiety reported by dental students [6] and 25% by medical students [7] since the beginning of pandemic.

Unfortunately, we tend to forget that mental disorders can occur in any person due to a pandemic, because of social isolation and loneliness. Depression is the most common one. It is estimated that about 350 million people are affected by this disease [8]. The number has significantly grown due to the COVID-19 pandemic. Many people experience depression on a daily basis, but are unaware that feeling worse, the lack of will to live, and frequent fatigue can be the basis for depression to develop [9]. An analysis of 1123 students in Poland showed that 76.96% of the participants manifested psychopathological symptoms [10]. A recent manuscript published in *Lancet* compared the prevalence of major depressive disorders and anxiety disorders before and during the COVID-19 pandemic. The authors showed that, before adjustment to the COVID-19 pandemic, the estimated global number of major depressive disorders was 193 million people, while after, it increased to 246 million people. A similar increase was found with anxiety disorders—298 million versus 374 million people [11]. Other studies show an increase in depressive and neurotic disorders during a pandemic, especially in students, so we wanted to see if this could be influenced by the method of learning and social isolation, in the form of reduced contact due to hybrid and online teaching. We analyzed the relationship between non-clinical (early) symptoms of depressed mood, personality traits, and coping strategies as well as whether the learning mode (online versus hybrid) differentiates the experiences of these early symptoms and coping strategies.

The following factors were analyzed: (1) the moods of students studying online and in hybrid form during the COVID-19 pandemic; (2) a correlation between non-clinical symptoms of low moods and coping styles in difficult and stressful situations; (3) the impact of the study mode (online or hybrid) on coping styles in stressful situations (see Table 1).

Two hypotheses were formulated:

**Hypothesis** **1** **(H1).** *There are correlations between non-clinical symptoms of a low mood or depression and coping strategies in difficult and stressful situations.*

**Hypothesis** **2** **(H2).** *There are significant differences in coping strategies between students learning online and in a hybrid way.*

## 2. Materials and Methods

### 2.1. Participants

The study comprised 114 university students (71 women and 43 men) aged 19 to 34 (M = 22.61, SD = 2.33), whose educational models changed from stationary to hybrid (56 students) or online (58 students) due to COVID-19 restrictions. All respondents gave their informed consent to participate in the study. Participation in the study was anonymous, voluntary, and not limited in time.

### 2.2. Measures and Procedures

Participants completed two online tools: (1) a questionnaire asking for demographic data, learning mode (Table 1), quality of life during the pandemic, and nonclinical symptoms of depression. The factor analysis using the principal components method was conducted, concerning questions about non-clinical symptoms of a depressed mood or depression. It enabled the final internal structure of the questionnaire to be obtained. Twenty-three questions with factor loadings greater than 0.40 were extracted. The scree plot criterion, which suggests the existence of a 5-factor solution, was used to determine the number of components:

FACTOR I_NEGATIVE PSYCHOLOGICAL STATE (Cronbach α = 0.848) included six questions: (4) have you experienced excessive fatigue in the last 6–12 months?—Factor load 0.409. (5) Have you experienced excessive psychological stress in the last 6–12 months?—Factor load 0.717. (6) During the past 6–12 months, have you experienced unreasonable anxiety?—Factor load 0.813. (7) Have you experienced unreasonable outbursts of anger in the last 6 to 12 months?—Factor load 0.628. (8) During the past 6–12 months, have you experienced unexplained sadness?—Factor load 0.618. (29) Do you feel you have poor mental toughness?—Factor load 0.717.

FACTOR II_NEGATIVE THOUGHTS (Cronbach α = 0.823) comprised five questions: (10) in the last 6 to 12 months, have you experienced any intrusive thoughts that you find difficult to get rid of?—Factor load 0.468. (11) During the past 6–12 months, have you ever wondered why you did not act differently in certain situations?—Factor load 0.567. (24) During the last 6–12 months, how often did you think back to a situation over which you no longer had any control?—Factor load 0.701. (31) Over the past 6–12 months, has your sense of loneliness and emptiness increased?—Factor load 0.611. (32) Over the last 6–12 months, has your sense of guilt or worthlessness increased?—Factor load 0.694.

FACTOR III_LOSS OF COGNITIVE ACTIVITY (Cronbach α = 0.891) comprised five questions: (19) over the last 6–12 months, have you noticed any increase in problems with concentration of attention?—Factor load 0.863. (20) Within the last 6–12 months, do you notice more intense problems with remembering/learning/assimilating new knowledge?—Factor load 0.821. (21) Over the last 6–12 months, do you experience more intense problems with motivation to act?—Factor load 0.814. (22) Over the last 6–12 months, do you experience more intense problems with decision-making?—Factor load 0.480. (23) How often during the last 6–12 months did you put off doing necessary but aversive tasks to the last moment?—Factor load 0.702.

FACTOR IV_SELF-ESTEEM (Cronbach α = 0.739) consisted of four questions: (26) I consider myself physically attractive—factor load 0.730. (27) I consider myself socially attractive—factor load 0.799. (28) I consider myself smart—factor load 0.723. (30) I consider myself responsible—factor load 0.459.

FACTOR V_ SUBSTANCE ABUSE (Cronbach α = 0.630) included three questions: (42) how often in the last 6–12 months have you used drugs or legal highs?—Factor load 0.698. (43) How often, during the last 6–12 months, did you use alcohol?—Factor load 0.457. (44) During the last 6—12 months, how often did you use sleeping pills, tranquilizers, or antidepressants?—Factor load 0.718.

Factor loadings were determined using Oblimin oblique rotation [7]. The extracted factors explain nearly 69% of the variance. Statistical data that define the characteristics of the KMO correlation matrix (0.827) and Bartlett’s sphericity test (χ^2^ = 1323.01; *p* < 0.001) indicate good data quality and correlations statistically different from 0. Respondents were asked to indicate the subjective importance of individual factors on a 4-point Likert scale (depending on the type of question with endpoints: minimum 1—not at all/definitely rarely/definitely not; maximum 4—definitely often/definitely yes).

(2) Brief-COPE Inventory by Carver [12], a Polish adaptation, Mini-COPE Inventory [13], was used to assess coping with stress. It consists of 28 statements; the respondent answers on a 4-point scale from 0 to 3, where 0 means: I almost never behave this way and 3 means: I almost always behave this way. The statements are grouped into 14 strategies (2 statements in each strategy): active coping, planning, seeking instrumental support, seeking emotional support, avoiding competitive actions, turning to religion, positive reevaluation and development, refraining from action, acceptance, focusing on and discharging emotions, denial, distraction, stopping action, using alcohol or other psychoactive substances, sense of humor. It is commonly used to measure dispositional coping.

The reliability of the original version reached, for most scales, a value close to α = 0.70 [14]. In the Polish adaptation, the reliability indices turned out to be much more varied and ranged from α = 0.32 (engaged in various activities) to 0.90 (turning to religion).

### 2.3. Statistical Analyses

The analyses were performed with IBM SPSS Statistics 26.0 for Windows. Data were summarized as percentages, mean, median, and standard deviations. The normality of the distribution was verified by the Kolmogorov–Smirnov test for the whole group (*n* > 100) and by the Shapiro–Wilk test for subgroups based on the learning mode: online or hybrid (*n* < 100) ( Table 2) [15].

Correlation analyses between non-clinical symptoms of depressed mood or depression and coping strategies were performed using the non-parametric rho-Spearman test. Due to the equal number of subjects in the compared groups (χ^2^ = 0.035, *p* = 0.851), homogeneity of variance was also tested. These two conditions, the equal number of subjects in the study groups, and homogeneity of variance confirmed by Levene’s test, justified the use of parametric statistical tests for hypothesis verification. Therefore, Student’s *t* test [12] will be used for further analysis. The condition of homogeneity of variance was not confirmed for only two factors (TAKING PSYCHOACTIVE SUBSTANCES and SELF BLAME). Therefore, the non-parametric Mann–Whitney U test for independent samples will be used for statistical analyses. The test results were treated as statistically significant for *p* < 0.05 [15].

## 3. Results

First, descriptive statistics were performed to enable the proper selection of statistical tests. As part of these analyses, the mean values, median, standard deviation, and values of Kolmogorov–Smirnov tests (in the case of analysis of results for the whole study group, *n* > 100) or Shapiro–Wilk (in the case of division into the online and hybrid learning group, *n* < 100) were determined. The data summarized in Table 2 indicate that the distributions obtained for the questionnaire on non-clinical symptoms of depressed moods or depression are consistent with “not rejected” normal distribution, only for factors I and III in the group of hybrid students, and factor II in both compared groups. This justifies the use of rho-Spearman statistics to study hypothesis I. For the 14 Mini-COPE inventory factors, the distributions obtained for all factors differ significantly from the normal distributions.

### Verification of Hypotheses

A correlation analysis based on the rho-Spearman test was performed to check correlations between non-clinical symptoms of a low mood or depression and coping strategies (Table 3).

An analysis of Table 3 reveals a number of significant correlations between non-clinical symptoms of a low mood or depression and coping strategies. However, most of them are very weak. Noteworthy are the moderate and strong correlations among:

- Negative psychological state and denial, suppression of competing activities, blaming oneself—moderate positive. In the group of students studying online: denial—moderate positive; suppression of competing activities—strong positive; whereas in the group of students studying in the hybrid mode: positive reinterpretation—strong negative, acceptance, use of emotional, social support -moderate negative, suppression of competing activities—moderate positive; blaming oneself—strong positive.
- Negative thoughts and focus on and venting of emotions, suppression of competing activities—moderate positive and blaming oneself—strong positive. In both groups of students: blaming oneself—strong positive; In addition, in the group of hybrid students: active coping, planning, acceptance, use of emotional, social support—moderate negative; positive reinterpretation—strong negative; suppression of competing activities—moderate positive.
- Loss of cognitive activity and focus on and venting of emotions, suppression of competing activities—moderate positive. In the group of students studying online: active coping—moderate negative; denial, suppression of competing activities—moderate positive. In both groups of students: focus on and venting of emotions—moderate positive. In the group of students studying in the hybrid mode: blaming oneself—moderate positive.
- Positive self-esteem and blaming oneself—moderate negative; active coping, positive reinterpretation, acceptance, use of emotional social support, use of instrumental social support—moderate positive. In both groups of students: active coping, positive reinterpretation—moderate positive. In the group of students studying in the hybrid mode: blaming oneself—strong negative; suppression of competing activities—moderate negative; use of instrumental social support—strong positive both moderate positive in the group of students studying online. In this group also: substance use—moderate negative.
- Stimulants and active coping, planning—moderate negative, suppression of competing activities—moderate positive; substance use—strong positive. Active coping was strong in the online learning group and moderated in the hybrid. In the group of students studying online: planning, acceptance—moderate negative; substance use—very strongly positive, while in the hybrid group—moderate. In both groups: suppression of competing activities—moderate positive.

**Figure 1:**
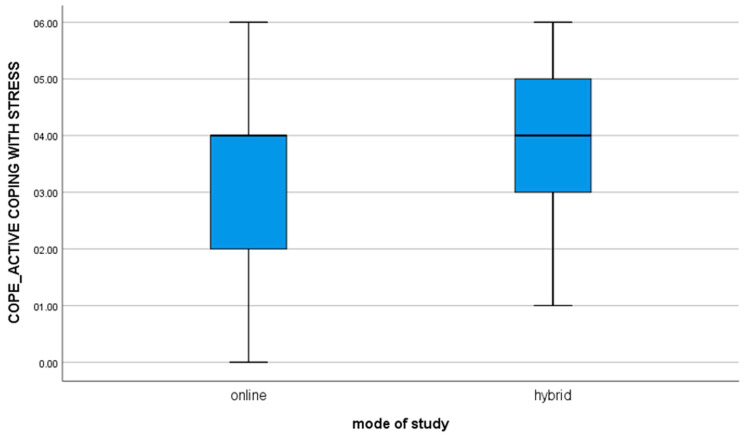
For the variable COPE_ACTIVE COPING WITH STRESS.

**Figure 2:**
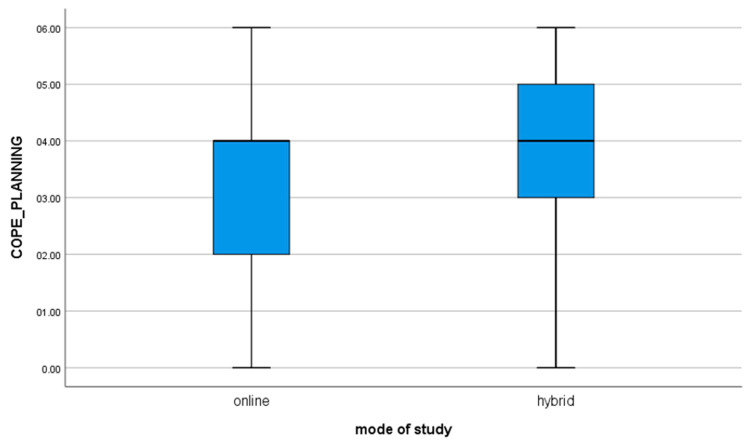
For the variable COPE_PLANNING.

**Figure 3:**
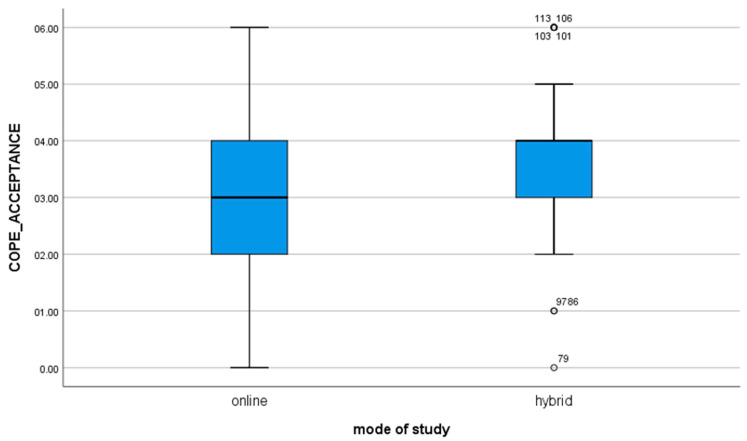
For the variable COPE_ACCEPTANCE.

**Figure 4:**
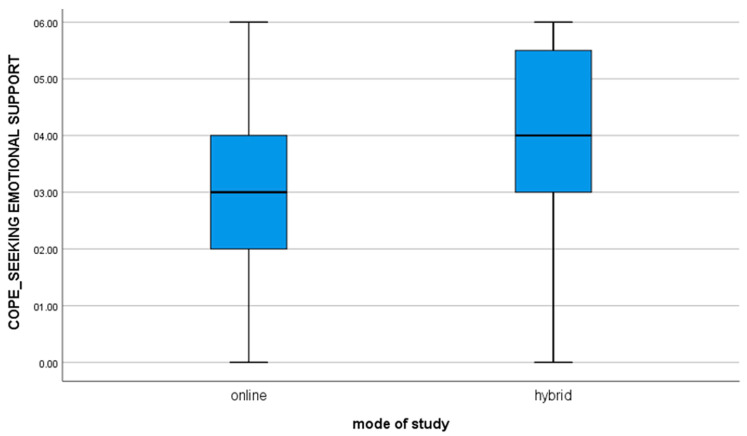
For the variable COPE_SEEKING EMOTIONAL SUPPORT.

**Figure 5:**
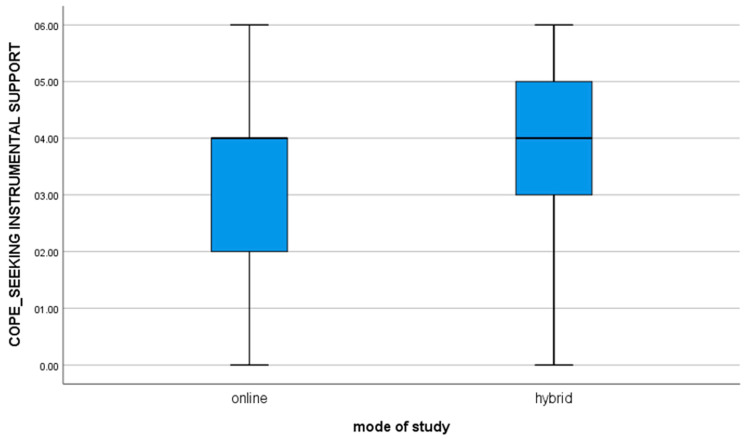
For the variable COPE_SEEKING INSTRUMENTAL SUPPORT.

**Figure 6:**
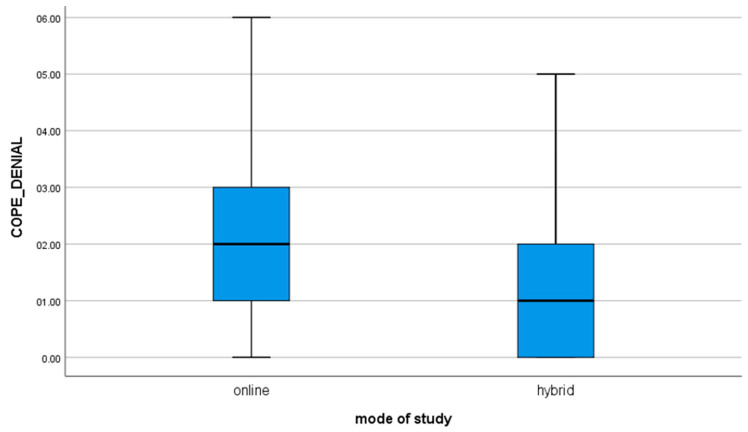
For the variable COPE_DENIAL.

To assess if the mode of study (online or hybrid) significantly differentiates the coping styles—*t*-statistic was used. It gave the following results:

1. for the variable COPE_ACTIVE COPING WITH STRESS, t(112) = −3.10; *p* = 0.002, the mean of the results in the group of online students is statistically significantly different from the results obtained by students studying in the hybrid mode (value of the mean difference: −0.82). The negative result shows that hybrid learners are significantly more likely to use active ways of coping with stressful situations as compared to online learners (Figure 1).

2. for the variable COPE_PLANNING, t(112) = −2.12; *p* = 0.036, the mean of the results in the group of students studying in the online mode is statistically significantly different from the results obtained by students studying in the hybrid mode (value of the mean difference: −0.55). The negative result shows that hybrid learners are significantly more likely to use self-planning strategies in stressful situations as compared to online learners (Figure 2).

4. for the variable COPE_ACCEPTANCE, t(112) = −2.68; *p* = 0.009, the mean of the results in the group of students studying in the online mode is statistically significantly different from the results obtained by students studying in the hybrid mode (value of the mean difference: −0.62). The negative result shows that hybrid learners are significantly more likely to accept the situation by learning how to live in it as compared to online learners (Figure 3).

7. for the variable COPE_SEEKING EMOTIONAL SUPPORT, t(112) = −3.13; *p* = 0.002, the mean of the results in the group of students studying online is significantly different from the results obtained by students studying hybrid (value of mean difference: −0.86). The negative result shows that hybrid learners are significantly more likely to seek encouragement, understanding, and support from others as compared to online learners (Figure 4).

8. for the variable COPE_SEEKING INSTRUMENTAL SUPPORT, t(112) = −3.01; *p* = 0.003, the mean of the results in the group of students studying online is significantly different from the results obtained by students studying hybrid (value of mean difference: −0.84). The negative result shows that hybrid learners are significantly more likely to seek and receive advice and help from others as compared to online learners (Figure 5).

10. for the variable COPE_DENIAL, t(112) = 2.71; *p* = 0.008, the mean of the results in the group of students studying online is significantly different from the results obtained by the students studying hybrid (value of mean difference: 0.68). The positive result shows that online learners are significantly more likely to reject the fact of a situation compared to hybrid learners (Figure 6).

## 4. Discussion

The previous pandemics occurred at times when mental health did not receive as much attention. Nonetheless, analyzing data following the Spanish flu pandemic in the 20th century, it is evident that infected individuals were significantly more likely than healthy individuals to report sleep disturbances, depression, mental distraction, dizziness, and difficulty in coping at work. In the U.S., an increase in suicide rates was reported in the years following the pandemic [16]. In addition, patients who recovered from the Spanish flu were more likely to report depression, neuropathy, and neurasthenia [17]. Moreover, the study conducted three years after the 2003 SARS outbreak noted moderate to severe symptoms of anxiety, depression, and post-traumatic stress disorder (PTSD) in approximately 20–30% of infected individuals. Furthermore, the risk of persistent symptoms and psychiatric disorders was higher in medical workers and those in quarantine [18]. Hence, it may be assumed that mental problems associated with the COVID-19 pandemic may persist for at least three years after the end of the pandemic.

The COVID-19 pandemic has a profound impact on all aspects of life. It may be associated with psychiatric symptoms in both adults and the pediatric population [19]. A study among the adult population in 2020 found that clinically significant psychiatric symptoms of anxiety, depression, distress, and PTSD were present in up to 36% of participants [18]. A study in China noted that about 20% of primary school students who experienced home quarantine reported anxiety and depressive symptoms [20]. Additionally, a study from Poland indicates that 24% of students have declared occurrences of suicidal thoughts since the beginning of the pandemic, whereas 19% of respondents reported results indicative of both anxiety and depressive disorders [21].

Moreover, any event that limits freedom, and forces individuals to change their current lifestyles, profoundly affects their psyches. Paul Harrison, Professor of Psychiatry at the University of Oxford, said, “People fear that people who have survived COVID-19 will be more likely to have mental health problems, and our findings show that this is likely”. According to the psychiatrist, health services must be prepared to provide care for patients after COVID-19 who develop mental health problems. In addition to SARS- COV 2 virus infection and fear for one’s health, self-isolation is a major cause of mental disorders. Therefore, it is essential to provide alternate teaching and working conditions to enable constant contact with other people. Based on our analysis and studies in other centers, this could reduce adverse effects.

Seeking evidence to support hypothesis 1 (H1): there are correlations between non-clinical symptoms of a low mood or depression and coping strategies in difficult and stressful situations. We conducted correlation analyses between the well-being of students and their subjectively assessed quality of life under COVID-19 pandemic conditions, and disclosed non-clinical symptoms of depression, or a low mood, and coping strategies. We found multiple significant correlations between symptoms of depressed moods and non-clinical symptoms of depression and coping strategies. To verify hypothesis 2 (H2)—that the mode of study significantly differentiates coping styles—the t-test was used. We found that differences between many adaptive coping strategies (such as active coping, planning, acceptance, seeking emotional or instrumental support), are more frequent in students studying in the hybrid mode, while those studying online are more likely to use non-adaptive strategies (e.g., denial).

Using these strategies likely helped them control negative thoughts or reduce negative mental states to a greater extent than students studying online. Students in this group also exhibited positive self-esteem, which corresponded with seeking emotional and instrumental support. Therefore, students in this group were less likely to blame themselves or stop doing things. Coping styles and perceived social support, emotional and instrumental, appear to contribute to the well-being of people [22].

Surprisingly, only the hybrid learning group showed an increased tendency to blame themselves for their negative psychological state. This might suggest a link to a sense of internal control (I am responsible for what I feel, and I need to do something about it), and might be related to a mode of social activity (I meet other people and my mood may be shared with them). These findings seem all the more plausible as self-blame co-occurred in this group, in conjunction with cognitive impairment. Cognitive impairment occurred in both groups, but it was combined with a cessation of activities in online students.

In contrast, non-adaptive coping strategies, such as denial, discharge, or cessation of actions, were revealed more frequently in the online study group in association with a negative psychological state. Although cessation of actions was revealed by students studying in the hybrid mode, this strategy seems to increase with the tendency to harbor negative thoughts about the self and the world.

Additionally, online students reported a higher propensity to use psychoactive substances, which was negatively associated with active coping strategies in stressful situations. Mheidly, Fares, and Fares [23] reported similar results, regarding the propensity of online learners to use non-adaptive strategies (such as alcohol consumption). Other studies found that subjective loneliness during lockdown rather than objective isolation was the strongest predictor of depression symptoms [24]. Moreover, a study in Poland showed that COVID-19-related anxiety was significantly more common in lonely individuals and in those of worse financial status [25]. The maladaptive coping mechanisms of those who fear viral infections were, in turn, highlighted by La Rosa et al. [26]. Studies conducted between COVID-19 waves confirm that the pandemic is a significant traumatic stressor leading to PTSD symptoms [27]. In regard to Italian university students and workers examined during the COVID-19 lockdown—24.2% reported depressive symptoms and 32.6% anxious symptoms [28]. Anxiety, fear, and worse quality of sleep during the pandemic increased cortisol levels, reduced melatonin synthesis, and caused changes in the biological rhythms [29]. Chronic stress, as during the COVID-19 pandemic, and subsequent malfunctioning of the hypothalamic-pituitary-adrenal axis, are involved in the pathogenesis of depression [30].

The differences in the study mode should be taken into consideration by university and school authorities. Opportunities to meet in a peer group during formal educational activities may foster adaptive, active coping strategies and, thus, reduce the rates of negative or depressed moods, and even non-clinical symptoms of depression. Due to the increase in digitization and a possible reduction of costs, there is a tendency to move towards online learning. However, the study indicates that this might have a destructive influence on mental health. The study by Rogowska AM et al. confirms psychological problems among students in Poland during the COVID-19 pandemic, in which 65% of respondents showed general anxiety disorders, whereas 56% experienced a high level of perceived stress. [31]. This calls for further studies on the impact of isolation on different university groups, as well as on healthcare professionals who work in the parent department or COVID units. Moreover, it seems worthy to test whether there is a difference in mental health between the two learning conditions and determine whether coping strategies play a significant role. However, certain limitations of the presented study should be acknowledged. Firstly, the group of respondents, the majority of whom were medical students, was relatively small and uniform, which might have had impact on the statistical diversity. In addition, an online questionnaire was solely implemented to record the responses of subjects; the extensive, detailed design of the inventory required constant focus from the participants; therefore, some of the responses might have been unreliable, i.e., caused by distraction and/or ennui.

## 5. Conclusions

A fully online study mode has a negative impact on the mental health of students. Students who learn in a hybrid mode are more likely to use active and positive coping strategies, which effectively help to control negative thoughts and/or reduce negative mental states. Non-adaptive coping strategies, such as denial, discharge, or cessation of actions were more frequently revealed in the online study group, in association with the negative psychological state. The online study group was characterized by a higher propensity to use psychoactive substances. Students have a strong negative view of fully remote studies, and they expressed their longing for a return to face-to-face contact learning. Systematic opportunities to meet in a peer group, as shown by the results of the hybrid learning group, might foster the use of adaptive, active coping strategies and, thus, reduce the rates of negative or depressed moods, and even non-clinical symptoms of depression. The COVID-19 pandemic will have significant psychological effects that will extend to the coming years; therefore, care seems to be of the utmost importance.

## Figures and Tables

**Table 1:** Demographic characteristics of participants (*n* = 114).

		*n*	%
***LEARNING MODE***	Online	58	50.9
	Hybrid	56	49.1
***FIELD OF STUDY***	Biotechnology	2	1.8
	Construction	2	1.8
	Economics	2	1.8
	English philology	2	1.8
	Finance and accounting	5	4.4
	Physiotherapy	2	1.8
	Computer science	3	2.6
	Medical	45	39.5
	Medical–dental	6	5.3
	Mathematics	2	1.8
	Pedagogy	3	2.6
	Law	6	5.3
	Psychology	2	1.8
	Transport	2	1.8
	Veterinary medicine	4	3.5
	Management	10	8.8
	Other (one person each from the faculties: banking and finance, electroradiology, electrotechnology, pharmacy, Iberian philology, geodesy, geoinformatics, software engineering, image communication, mechanics and mechanical engineering, horticulture, early childhood and preschool pedagogy, agriculture, international relations, physical education, zootechnics)	16	14.4
***PLACE***	Village	22	19.3
***OF RESIDENCE***	Small town	6	5.3
	Middle town	14	12.3
	Large city	72	63.2
***COVID-19 INFECTION***	No	52	45.6
	Yes	32	28.1
	Don’t know	30	26.3
***QUESTIONNAIRE INFORMATION***		***n***	**%**
*Adaptation*	Definitely not	6	5.3
*to the conditions*	No	44	38.6
	Yes	49	43
	Definitely yes	15	13.2
*Mental deterioration*	Definitely not	11	9.6
	No	48	42.1
	Yes	47	41.2
	Definitely yes	8	7
*Anxiety about education/work*	Not at all or definitely rarely	16	14
	Seldom	49	43
	Frequently	43	37.7
	Definitely frequently	6	5.3
*Study/work mode assessment*	Definitely negative	16	14
	Negative	49	43
	Positive	43	37.7
	Definitely positive	6	5.3
*More time to study*	Definitely not	14	12.3
	No	37	32.5
	Yes	48	42.1
	Definitely yes	10	8.8
*Back to pre-pandemic mode*	Definitely not	2	1.8
	No	30	26.3
	Yes	51	44.7
	Definitely yes	31	27.2
*More time for yourself/hobby*	Definitely not	8	7
	No	38	33.3
	Yes	55	48.2
	Definitely yes	13	11.4
*Systematic sporting activities*	Definitely not	18	15.8
	No	48	42.1
	Yes	32	28.1
	Definitely yes	16	14
*Meeting with friends*	Definitely not	17	14.9
*more often in a home setting*	No	52	45.6
	Yes	28	24.6
	Definitely yes	17	14.9
*Thinking about threats*	Not at all or definitely rarely	36	31.6
*to humanity and the world*	Seldom	40	35.1
	Frequently	32	28.1
	Definitely frequently	6	5.3
*Thinking about your own future*	Not at all or definitely rarely	7	6.1
	Seldom	12	10.5
	Frequently	68	59.6
	Definitely frequently	27	23.7
*A positive view of your own future*	Definitely not	1	0.9
	No	25	21.9
	Yes	74	64.9
	Definitely yes	14	12.3
*A positive view of the world*	Definitely not	4	3.5
	No	54	47.4
	Yes	47	41.2
	Definitely yes	9	7.9
*Anxiety for loved ones*	Not at all or definitely rarely	16	14
	Seldom	43	37.7
	Frequently	46	40.4
	Definitely frequently	9	7.9
*Anxiety for your future*	Not at all or definitely rarely	18	15.8
	Seldom	43	37.7
	Frequently	48	42.1
	Definitely frequently	5	4.4

**Table 2:** Descriptive statistics for factors in the coping styles variable and non-clinical symptoms of depressed mood or depression.

QUESTIONNAIRE						
Variable (*n* = 114)		*M*	*MD*	*SD*	Normality of Distribution Tests	
***Negative***	Global	14.14	14	4.07	*Z (p)*	0.094 (0.016)
***psychological state***	Online	13.71	14	3.95	*W (p)*	0.955 (0.031)
	Hybrid	14.6	15	4.17	*W (p)*	0.974 (0.278)
***Negative thoughts***	Global	12.2	12	3.11	*Z (p)*	0.099 (0.009)
	Online	11.93	12	2.81	*W (p)*	0.975 (0.280)
	Hybrid	12.49	13	3.4	*W (p)*	0.964 (0.099)
***Loss of cognitive activity***	Global	13.63	14	4.05	*Z (p)*	0.102 (0.006)
	Online	12.95	13.5	4.15	*W (p)*	0.956 (0.036)
	Hybrid	14.34	15	3.84	*W (p)*	0.958 (0.051)
***Self-esteem***	Global	11.59	12,00	1.92	*Z (p)*	0.186 (<0.001)
	Online	11.22	12	1.78	*W (p)*	0.930 (0.003)
	Hybrid	11.44	12	1.99	*W (p)*	0.943 (0.011)
***Substance abuse***	Global	4.87	4	1.69	*Z (p)*	0.218 (<0.001)
	Online	5.03	4.5	2.03	*W (p)*	0.850 (<0.001)
	Hybrid	4.69	4	1.21	*W (p)*	0.889 (<0.001)
*MINI-COPE*						
Variable (*n* = 114)		*M*	*MD*	*SD*	Normality of distribution tests	
***Active coping***	Global	3.8	4	1.46	*Z (p)*	0.213 (<0.001)
***with stress***	Online	3.4	4	1.51	*W (p)*	0.921 (0.001)
	Hybrid	4.21	4	1.29	*W (p)*	0.904 (<0.001)
***Planning***	Global	3.77	4	1.14	*Z (p)*	0.222 (<0.001)
	Online	3.5	4	1.45	*W (p)*	0.932 (0.003)
	Hybrid	4.05	4	1.33	*W (p)*	0.903 (<0.001)
***Positive revaluation***	Global	3.24	3	1.4	*Z (p)*	0.190 (<0.001)
	Online	3.03	3	1.39	*W (p)*	0.905 (<0.001)
	Hybrid	3.45	3.5	1.39	*W (p)*	0.935 (0.005)
***Acceptance***	Global	3.56	4	1.26	*Z (p)*	0.224 (<0.001)
	Online	2.26	3	1.19	*W (p)*	0.908 (<0.001)
	Hybrid	3.87	4	1.26	*W (p)*	0.881 (<0.001)
***Sense of humor***	Global	1.97	2	1.31	*Z (p)*	0.150 (<0.001)
	Online	1.9	2	1.37	*W (p)*	0.923 (0.001)
	Hybrid	2.05	2	1.25	*W (p)*	0.915 (0.001)
***Turning to religion***	Global	2.05	2	1.94	*Z (p)*	0.206 (<0.001)
	Online	1.95	2	1.82	*W (p)*	0.874 (<0.001)
	Hybrid	2.16	2	2.07	*W (p)*	0.856 (<0.001)
***Seeking emotional support***	Global	3.63	4	1.15	*Z (p)*	0.165 (<0.001)
	Online	3.21	3	1.42	*W (p)*	0.936 (0.004)
	Hybrid	4.07	4	1.52	*W (p)*	0.904 (<0.001)
***Seeking instrumental support***	Global	3.53	4	1.55	*Z (p)*	0.215 (<0.001)
	Online	3.12	4	1.39	*W (p)*	0.889 (<0.001)
	Hybrid	3.96	4	1.59	*W (p)*	0.913 (0.001)
***Getting engaged***	Global	3.34	3	1.36	*Z (p)*	0.168 (<0.001)
***in various activities***	Online	3.15	3	1.32	*W (p)*	0.940 (0.006)
	Hybrid	3.53	4	1.39	*W (p)*	0.942 (0.009)
***Denial***	Global	1.63	2	1.38	*Z (p)*	0.184 (<0.001)
	Online	1.96	2	1.51	*W (p)*	0.915 (0.001)
	Hybrid	1.28	1	1.14	*W (p)*	0.873 (<0.001)
***Venting emotions***	Global	3.23	3	1.23	*Z (p)*	0.189 (<0.001)
	Online	3.1	3	1.16	*W (p)*	0.923 (0.001)
	Hybrid	3.36	3.5	1.28	*W (p)*	0.922 (0.001)
***Taking psychoactive substances***	Global	1.2	0	1.73	*Z (p)*	0.335 (<0.001)
	Online	1.55	0	2	*W (p)*	0.755 (<0.001)
	Hybrid	0.84	0	1.32	*W (p)*	0.680 (<0.001)
***Resignation***	Global	1.87	2	1.45	*Z (p)*	0.183 (<0.001)
	Online	2.09	2	1.47	*W (p)*	0.917 (0.001)
	Hybrid	1.64	2	1.41	*W (p)*	0.890 (<0.001)
***Self-blame***	Global	2.89	3	1.56	*Z (p)*	0.190 (<0.001)
	online	2.67	2.5	1.35	*W (p)*	0.938 (0.005)
	hybrid	3.12	3.5	1.74	*W (p)*	0.926 (0.002)

*M*—mean; *MD*—median; *SD*—standard deviation; *Z*—Kolmogorov–Smirnov test for *n* > 100; *W*—Shapiro–Wilk test for *n* < 100.

**Table 3:** Correlations between non-clinical symptoms of a low mood or depression and coping strategies in difficult and stressful situations for general (*n* = 114) and online *(n* = 58) and hybrid learning group *(n* = 56).

SPEARMAN’S RHO *(p)*			QUESTIONNAIRE				
			Negative Psychological State	Negative Thoughts	Loss of Cognitive Activity	Self-Esteem	Substance Abuse
**COPE**	*Active coping with stress*	global	−0.214 *	−0.143	−0.158	0.376 **	−0.408 **
		online	−0.262 *	−0.019	−0.328 *	0.387 **	−0.517 **
		hybrid	−0.224	−0.313 *	−0.086	0.331 *	−0.302 *
	*Planning*	global	−0.167	−0.143	−0.140	0.288 **	−0.301 **
		online	−0.114	0.008	−0.291 *	0.257	−0.328 *
		hybrid	−0.276 *	−0.318 *	−0.041	0.274 *	−0.296 *
	*Positive revaluation*	global	−0.245 **	−0.242 **	−0.116	0.420 **	−0.195 *
		online	0.040	0.031	−0.103	0.417 **	−0.284 *
		hybrid	−0.554 **	−0.501 **	−0.182	0.388 **	−0.095
	*Acceptance*	global	−0.144	−0.127	−0.047	0.312 **	−0.267 **
		online	−0.012	0.082	0.010	0.285 *	−0.346 **
		hybrid	−0.337 *	−0.368 **	−0.158	0.268 *	−0.194
	*Sense of humor*	global	−0.008	−0.019	0.066	0.128	0.057
		online	0.066	0.148	0.048	0.121	0.019
		hybrid	−0.104	−0.190	0.081	0.125	0.105
	*Turning to religion*	global	0.046	0.014	0.025	0.126	0.007
		online	0.229	0.112	0.133	0.170	−0.055
		hybrid	−0.135	−0.069	−0.095	0.104	0.063
	*Seeking emotional support*	global	−0.236 *	−0.239 *	−0.094	0.447 **	−0.171
		online	−0.187	−0.037	−0.074	0.289 *	−0.177
		hybrid	−0.341 *	−0.481 **	−0.192	0.527 **	−0.192
	*Seeking instrumental support*	global	−0.054	−0.108	0.013	0.474 **	−0.126
		online	0.035	0.058	−0.040	0.305 *	−0.226
		hybrid	−0.178	−0.299 *	−0.009	0.574 **	−0.029
	*Getting engaged in various activities*	global	0.028	0.088	0.064	0.056	−0.056
		online	−0.019	0.043	−0.149	0.222	−0.136
		hybrid	0.043	0.095	0.248	−0.134	0.037
	*Denial*	global	0.328 **	0.231 *	0.220 *	−0.062	0.184
		online	0.494 **	0.254	0.321 *	0.095	0.239
		hybrid	0.227	0.312 *	0.204	−0.157	0.115
	*Venting emotions*	global	0.257 **	0.243 **	0.346 **	0.015	0.187 *
		online	0.282 *	0.296 *	0.368 **	0.125	0.222
		hybrid	0.205	0.151	0.304 *	−0.134	0.155
	*Taking psychoactive substances*	global	0.057	0.022	0.084	−0.275 **	0.651 **
		online	0.112	0.018	0.216	−0.372 **	0.754 **
		hybrid	0.032	0.071	−0.028	−0.139	0.498 **
	*Resignation*	global	0.405 **	0.283 **	0.302 **	−0.285 **	0.346 **
		online	0.525 **	0.177	0.466 **	−0.112	0.392 **
		hybrid	0.331 *	0.441 **	0.196	−0.442 **	0.314 *
	*Self-blame*	global	0.416 **	0.626 **	0.289 **	−0.419 **	0.149
		online	0.198	0.619 **	0.141	−0.267 *	0.213
		hybrid	0.600 **	0.623 **	0.423 **	−0.621 **	0.107

**. Correlation significant at 0.01 level (two-sided). *. Correlation significant at the 0.05 level (two-sided). Explanations: Questionnaire FACTOR I_Negative psychological state; FACTOR II_Negative thoughts; FACTOR III_Loss of cognitive activity; FACTOR IV_Self-esteem; FACTOR V_Substance abuse.

## Data Availability

The data that support the findings of this study are available upon reasonable request from the corresponding author. The data are not publicly available due to privacy.
